# Post-traumatic stress disorder among workers in Brazil: a narrative review

**DOI:** 10.47626/1679-4435-2025-1401

**Published:** 2025-07-13

**Authors:** Bruna Roberta Muntanelli, Marcos Felipe Bom Sampaio, Sérgio Roberto de Lucca, Marcia Bandini, Ivan Dieb Miziara, João Silvestre Silva-Junior

**Affiliations:** 1 Hospital das Clínicas, Faculdade de Medicina da Universidade de São Paulo (FMUSP), São Paulo, SP, Brazil; 2 Departamento de Medicina Legal, Bioética, Medicina do Trabalho e Medicina Física e Reabilitação, Faculdade de Medicina, FMUSP, São Paulo, SP, Brazil; 3 Departamento de Saúde Coletiva, Universidade Estadual de Campinas, Campinas, SP, Brazil

**Keywords:** stress disorders, post-traumatic, occupational health, mental health, transtornos de estresse pós-traumático, saúde ocupacional, saúde mental

## Abstract

Post-traumatic stress disorder is a complex psychiatric condition that can develop after exposure to traumatic events such as wars, natural disasters, or workplace violence. This study aims to conduct a review of the prevalence and contributing factors of post-traumatic stress disorder among Brazilian workers. A literature search was performed in the PubMed and SciELO databases between February and June 2023. The search focused on quantitative studies addressing post-traumatic stress disorder in Brazilian workers, using terms related to post-traumatic stress disorder, occupational exposure, and health aspects. Only studies involving Brazilian residents with post-traumatic stress disorder as an outcome were included, while reviews, case reports, and qualitative studies were excluded. Of the 12 studies initially identified, 11 met the inclusion criteria. Firefighters had the highest prevalence of post-traumatic stress disorder, ranging from 6.9% to 37.9%, followed by health care workers — especially nurses — and military police officers, who also showed considerable rates. Work-related factors associated with an increased risk of post-traumatic stress disorder included lower occupational ranks or higher job demands, long working hours, repeated exposure to traumatic events, lack of personal protective equipment, and exposure to hazardous materials. The study identified common occupational risk factors across various professions that contribute to the development of post-traumatic stress disorder. These findings underscore the need for targeted interventions aimed at improving working conditions and strengthening mental health support for workers.

## Abstract (pt)

O transtorno de estresse pós-traumático é uma condição psiquiátrica complexa que pode se desenvolver após a exposição a eventos traumáticos, como guerras, desastres naturais e violência relacionada ao trabalho. O estudo tem como objetivo realizar uma revisão sobre a prevalência e os fatores contribuintes para o transtorno de estresse pós-traumático entre trabalhadores brasileiros. Foi realizada uma revisão bibliográfica utilizando as bases de dados PubMed e SciELO entre fevereiro e junho de 2023. A pesquisa incluiu estudos quantitativos sobre transtorno de estresse pós-traumático entre trabalhadores no Brasil, utilizando termos relacionados ao transtorno de estresse pós-traumático, exposição ocupacional e aspectos de saúde. Apenas estudos envolvendo residentes brasileiros com desfechos de transtorno de estresse pós-traumático foram incluídos, enquanto revisões, relatos de caso e estudos qualitativos foram excluídos. Dos 12 estudos inicialmente selecionados, 11 foram incluídos na revisão final. Os bombeiros apresentaram a maior prevalência de transtorno de estresse pós-traumático, com taxas variando de 6,9% a 37,9%, enquanto trabalhadores da saúde, particularmente enfermeiros, e policiais militares também apresentaram taxas significativas. As principais variáveis relacionadas ao trabalho associadas ao aumento do risco de transtorno de estresse pós-traumático incluíram patentes mais baixas ou maiores demandas de trabalho, longas jornadas, exposição repetida a eventos traumáticos, falta de equipamentos de proteção individual e exposição a materiais perigosos. O estudo identificou fatores comuns relacionados ao trabalho em diversos grupos profissionais que contribuem para a incidência de transtorno de estresse pós-traumático. Esses achados sugerem a necessidade de intervenções direcionadas às condições de trabalho e ao suporte em saúde mental.

## INTRODUCTION

Post-traumatic stress disorder (PTSD) is a complex and debilitating psychiatric condition that can develop following direct or indirect exposure to traumatic events such as war, sexual assault, serious accidents, natural disasters, or violence occurring in domestic, public, or workplace settings, including during work-related activities.^1^ The experience or witnessing of such events has a significant impact on the lives of affected individuals, compromising their psychological well-being as well as their social and occupational functioning.^1^

The diagnostic criteria for PTSD include intrusive symptoms, avoidance behaviors, mood alterations, and hyperarousal following exposure to a traumatic event or a life-threatening situation.^1^ Intrusive symptoms involve recurrent and distressing memories of the traumatic event, nightmares, flashbacks, and intense physical reactions. Avoidance reactions may involve sweating and tachycardia, as well as conscious efforts to suppress memories of the event and associated places. Mood alterations include persistent negative thoughts about oneself or others, while hyperactivity (hyperarousal) is associated with a constant state of alertness, sleep disturbances, irritability, and difficulty concentrating.^1^

The prevalence of PTSD varies depending on the context and the vulnerability of a country or region. In Brazil, the estimated prevalence is approximately 6.1% in the general population.^2^ Workers engaged in occupations with higher exposure to traumatic and repetitive situations, rescue operations, and emergency response are more vulnerable to developing PTSD. This includes police officers, firefighters, and emergency health care professionals.^3^

According to Brazil’s List of Work-Related Diseases,^4^ “post-traumatic stress disorder” (ICD-10 F43.1) may be associated with psychosocial factors at work related to: organizational management; the organizational context of the work; the nature of social relationships in the workplace; the content of work tasks; the physical work environment; person-task interaction; working hours; workplace violence, moral or sexual harassment; discrimination at work; or exposure to life-threatening situations and trauma in the workplace.

Because of the importance of providing treatment for individuals affected by these events and the need to develop intervention and prevention strategies, it is essential to consider the epidemiology of PTSD in specific occupational contexts. This study aims to present a literature review on PTSD among Brazilian workers.

## METHODS

This is a documentary study based on a literature review of articles published in scientific journals indexed in the PubMed and SciELO databases. The search strategy in PubMed was based on the following search string: [“Stress Disorders, Post-Traumatic” AND ((“work”) OR “occupational” AND (“exposure” OR “health”)) AND “Brazil”]. For the SciELO database, the search string was [“Transtornos de Estresse Pós-Traumáticos” AND (ocupacional OR trabalho)]. Searches in both databases were conducted between February and June 2023.

**Figure 1:**
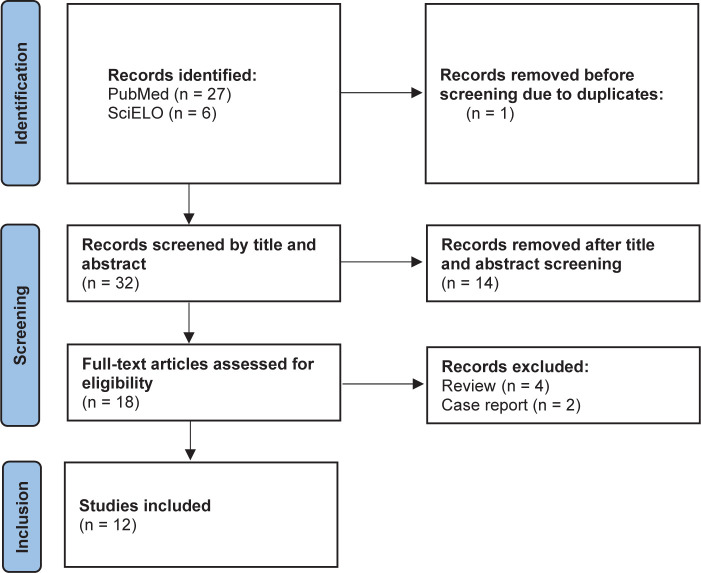
Preferred Reporting Items for Systematic Reviews and Meta-Analyses (PRISMA) flowchart of study selection from database search.

Only quantitative studies involving data from workers residing in Brazil, with PTSD as an outcome, were included. Quantitative studies provide numerical and objective data on the relationship between variables, allowing for a more precise measurement of the strength and direction of associations between predictive factors and outcomes. There were no time restrictions or limitations regarding the participants’ occupations. Publications in English, Spanish, and Portuguese were considered. Exclusion criteria included review articles, case reports, and studies that were primarily qualitative. Figure 1 presents the flowchart based on the Preferred Reporting Items for Systematic Reviews and Meta-Analyses (PRISMA) for the identification and selection of articles.

## RESULTS AND DISCUSSION

Of the 12 studies initially selected, one was excluded for not addressing work-related aspects, focusing solely on clinical outcomes in the general population.^5^ As a result, 11 articles were included in this review, of which 10 were cross-sectional and one was longitudinal. In all 11 studies analyzed, PTSD diagnosis was based on participants’ self-reports. Nine studies used the PTSD Checklist for the Diagnostic and Statistical Manual of Mental Disorders, 5th Edition (PCL-5)^6^-^14^; one study employed the Structured Clinical Interview for DSM-IV PTSD Module (SCID-PTSD)^15^; and another used the Impact of Events Scale – Revised (IES-R).^16^

Discussion is organized in Chart 1 according to each occupational group.

**Chart 1:** Summary of the main characteristics of studies on PTSD selected for the review

Authors	Year of publication	Design	Location	Data collection year	Participants	How PTSD was defined
Berger et al.^6^	2007	Cross-sectional	Rio de Janeiro – RJ	2003-2004	234 ambulance rescuers	Self-report (PCL-5)
Maia et al.^7^	2007	Cross-sectional	Goiás	2004	157 policy officers	Self-report (PCL-5)
Maia et al.^8^	2011	Cross-sectional	Goiás	2005	212 policy officers	Self-report (PCL-5)
Monteiro et al.^15^	2013	Cross-sectional	Rio dos Sinos – RS	2011	27 firefighters	Self-report (SCID-PTSD)
Lima et al.^9^	2015	Cross-sectional	Belo Horizonte – MG	2011	711 firefighters	Self-report (PCL-5)
Januário et al.^16^	2017	Cross-sectional	Divinópolis – MG	2014-2016	61 nursing professionals	Self-report (IES-R)
Campos et al.^10^	2021	Cross-sectional	Rio de Janeiro	2017-2019	3577 policy officers	Self-report (PCL-5)
Vasconcelos et al.^11^	2021	Longitudinal	Minas Gerais	2014 and 2017	501 firefighters	Self-report (PCL-5)
Bertolazi et al.^12^	2022	Cross-sectional	Santa Maria – RS	2013-2014	180 firefighters	Self-report (PCL-5)
Machado et al.^13^	2023	Cross-sectional	Various locations in Brazil	2020	941 health care professionals	Self-report (PCL-5)
Monteiro et al.^14^	2023	Cross-sectional	Metropolitan region of Belém – PA	2017-2019	30 firefighters	Self-report (PCL-5)

IES-R = Impact of Events Scale – Revised; PCL-5 = Post-traumatic Stress Disorder Checklist for the Diagnostic and Statistical Manual of Mental Disorders, 5th Edition (DSM-5); SCID-PTSD = Structured Clinical Interview for DSM-IV PTSD Module; PTSD = post-traumatic stress disorder.

### FIREFIGHTERS

The firefighter category was the most frequently studied regarding PTSD, with four studies.

Bertolazi et al.^12^ interviewed 180 firefighters involved in the rescue of victims from the Kiss nightclub fire (Santa Maria, RS), which resulted in 242 deaths and 636 injuries in 2013. The study was published nearly nine years after data collection due to the challenge of separating the impact of PTSD developed by the accident victims from its influence on the firefighter population. This limitation affected the objectivity of determining the profession’s exclusive contribution to PTSD symptoms. The only occupational variable associated with increased PTSD prevalence identified was work shifts. The authors argue that disruption of the circadian cycle due to night shifts undermines mental health, predisposing individuals to the development and/or worsening of the disorder. They emphasize the need for interventions focused on shift management to mitigate these adverse effects.

Monteiro et al.,^15^ also in the state of Rio Grande do Sul, but in the city of Rio dos Sinos, interviewed 27 firefighters to investigate their mental health and working conditions in southern Brazil. The findings showed that none of the participants met the full diagnostic criteria for PTSD.

Lima et al.^9^ conducted a study with 711 firefighters in Belo Horizonte, MG, and found a PTSD prevalence of 6.9% among participants. The main occupational factors associated with PTSD were high job demands and repeated exposure to traumatic events during daily work activities. This prevalence is comparable to rates observed in other emergency professional groups but slightly higher than that of the general Brazilian population.^2^

The study by Vasconcelos et al.^11^ investigated a prospective cohort of 312 novice firefighters over 2 years, aiming to identify occupational factors contributing to the development of PTSD symptoms. The results showed a significant increase in PTSD symptoms after 2 years of service, with key occupational stressors including low social support, lack of job control, high job demands, and frequent exposure to traumatic events identified as critical determinants.

These findings are consistent with those reported in the review by Almeida,^17^ which revealed a substantially higher prevalence of PTSD in this population compared to the general population. This study also identified lack of social support and high job demands as significant predictors of PTSD among firefighters. These results highlight the urgent need for preventive interventions and psychological support programs to mitigate the adverse impacts of working conditions on the mental health of firefighters and other emergency professionals.

### HEALTH CARE PROFESSIONALS

In this population group, two cross-sectional studies were selected for analysis.

The study by Machado et al.^13^ surveyed 941 health care professionals nationwide — including physicians, physical therapists, and nursing staff — during the challenging period of the COVID-19 pandemic in 2020. The findings showed that nursing technicians, who occupy one of the lowest ranks in the health care hierarchy, particularly young female professionals, had the highest prevalence of PTSD, accounting for 37.9% of cases. In other words, not only age and gender were statistically significant, but professional category also played a relevant role. Lower-ranking positions are likely to face more demanding working conditions in terms of workload and job strain.

In addition, the authors highlight another work-related variable that played a significant role in the development of PTSD: the availability — or lack — of personal protective equipment (PPE) during job performance. According to the study, the lack of PPE at the height of the pandemic placed certain groups of health care workers in a position of fragility and imminent vulnerability when facing a pathogenic agent that was still not well understood at the time and for which no vaccine was available, at least in Brazil. This fragility and vulnerability, interpreted as stressors, were reflected in the data: 33% of respondents diagnosed with PTSD reported not having access to PPE, while only 18.8% of those with PTSD reported having had adequate access to protective equipment. The shortage of PPE predisposed workers to more traumatic events and increased the prevalence of PTSD among these respondents.^13^

The second study, by Januário et al.,^16^ conducted between 2014 and 2016 in the city of Divinópolis, MG, with a sample of 61 nursing staff members, found results similar to those reported by Machado et al.^13^ One in five workers (19.6%) who experienced recurrent occupational exposure to biological material eventually developed PTSD.

### AMBULANCE RESCUERS

Only one article in the entire review focused on the ambulance rescuer population: the study by Berger et al.^6^ Interviewing 234 of these workers in Rio de Janeiro between 2003 and 2004, the authors aimed to compare the prevalence of partial PTSD with fully symptomatic PTSD. However, they did not assess which work-related variables might contribute to the development or worsening of the disorder. The prevalence of PTSD found in this population was 15%, nearly three times higher than the rate reported in the general Brazilian population.^2^ This high prevalence highlights the vulnerability of these professionals to work-related traumatic events and underscores the need for effective intervention and prevention strategies.

Evidence-based interventions emphasize the importance of psychological support programs and training for rescuers, who are consistently exposed to stressful events. These initiatives aim not only to mitigate the adverse impacts of psychological trauma but also to strengthen these professionals’ ability to manage high-stress situations more effectively and adaptively.^18^

### MILITARY POLICE OFFICERS

The study by Campos et al.^10^, which included the largest number of participants among the selected studies, examined a population of 3,577 members of the Military Police of Rio de Janeiro using a cross-sectional design. This population showed a PTSD prevalence ranging from 13% to 19% during the study period, from 2017 to 2019. These rates are very similar to those observed among ambulance rescuers (15%)^6^ and higher than the national average.^2^ The authors found that working hours, rank within the corporation, and job assignment were the work-related variables that directly influenced the development or worsening of PTSD. The study concluded that lower-ranking officers, who often have longer working hours — even those assigned to administrative duties — are the subgroups within the military police most affected by this mental disorder.

The study conducted by Monteiro et al.^14^ examined PTSD in 30 military police officers who sustained gunshot wounds in the Metropolitan Region of Belém, state of Pará. The results indicated that 36.67% of the injured officers were at risk of developing PTSD, with 20% classified as partial PTSD and 16.67% as full PTSD. Officers at greater risk were predominantly male, held the rank of corporal, and had between 10 and 15 years of active service. They performed operational duties and were often injured in situations such as attempted robbery or homicide. The study also found that these officers tended to have longer hospitalization periods, experienced more physical sequelae, reported poorer mental health, and received less psychosocial support from the military institution.

The study by Maia et al.^8^ analyzed a cross-sectional sample of 212 active-duty police officers to identify predictors of PTSD symptom severity. The results showed that negative affect, length of service, frequency of exposure to critical incidents, peritraumatic dissociation, and lack of social support were significant predictors of PTSD symptom severity.

## CONCLUSIONS

This review identified similar work-related variables across different groups of participants that may play a consistent role in the prevalence of PTSD within their respective populations. Variables such as *rank within the organization* and *job assignment* among military police officers, *professional hierarchy* among health care workers, and *job demands* among firefighters consistently revealed that the lower the hierarchical level—or the higher the job demands—the greater the prevalence of PTSD among participants in each occupational group.

Additionally, factors such as *long working hours* among military police officers, *repeated exposure to traumatic events* among firefighters and military police officers, *lack of PPE* among health care workers, and *recurrent occupational exposure to hazardous biological material* among nursing staff highlighted the significant similarity in how these working conditions influence the incidence of PTSD, regardless of the profession.

As a suggestion for future research, it would be valuable to explore how these patterns manifest in different cultural and national contexts, assessing how work-related variables impact the incidence of PTSD in various countries and cultures. Such studies could support the development of systemic and targeted strategies aimed at providing adequate support to improve the mental health and well-being of workers.
